# Effect of Resin Parameters on the Consistency and Mechanical Properties of Ultra-High-Molecular-Weight Polyethylene Fiber

**DOI:** 10.3390/polym17081109

**Published:** 2025-04-19

**Authors:** Cheng Yan, Tiantian Yan, Tianhong Dong, Mingxin Xia, Yumin Xia, Yong He

**Affiliations:** 1 State Key Laboratory for Modification of Chemical Fibers and Polymer Materials, College of Material Science and Engineering, Donghua University, Shanghai 201620, China; 2 LIANYUNGANG SHENTE HIGH-TECH MATERIALS CO., LTD., Lianyungang 222000, China; 3 Innovation Center for Textile Science and Technology, Donghua University, Shanghai 201620, China

**Keywords:** UHMWPE, molecular weight, particle size

## Abstract

Maintaining the consistency of linear density in ultra-high-molecular-weight polyethylene (UHMWPE) fiber has been a critical challenge in the production of UHMWPE fibers. However, there has been limited research focusing on the impact of UHMWPE resin parameters on the consistency in fiber linear density. In this study, a series of UHMWPE fibers were produced through wet spinning using UHMWPE resins with varying parameters. The effects of molecular weight, molecular weight distribution, particle size, and particle size distribution of UHMWPE resins on the consistency of linear density and the mechanical properties of UHMWPE fibers were systematically investigated. The experimental findings revealed that narrowing the molecular weight distribution and particle size distribution of ultra-high molecular weight polyethylene (UHMWPE) resin precursors significantly enhanced the consistency of resultant UHMWPE fibers, concurrently improving their tensile strength and elastic modulus. Notably, while the absolute molecular weight of the resin demonstrated no statistically significant correlation with fiber consistency, an optimal molecular weight range was identified to maximize the mechanical performance of UHMWPE fibers. Specifically, fibers synthesized from resin precursors within this molecular weight window exhibited peak values in both strength and modulus, suggesting a critical balance between molecular chain entanglement and processability.

## 1. Introduction

Ultra-high-molecular-weight polyethylene (UHMWPE) fiber, characterized by its exceptionally high molecular weight, exhibits remarkable properties [1,2,3], including high strength, impact resistance, wear resistance, self-lubrication, chemical resistance, and low-temperature resilience. These advantageous characteristics render UHMWPE fibers extensively utilized in critical applications such as ballistic protection, riot control measures, and high-performance ropes and cables. The poor thermal resistance and anti-creep performance of ultra-high-molecular-weight polyethylene (UHMWPE) fibers can be enhanced through physical and chemical modification methods. Fiber consistency critically influences product performance, particularly in protective applications. Inconsistent molecular weight distribution and particle size in precursor resins may lead to structural defects during processing, resulting in stress concentration points and reduced ballistic protection efficiency.

The properties of UHMWPE fibers are predominantly governed by the physicochemical characteristics of their precursor resin materials. The catalyst and process of UHMWPE polymerization significantly influence the parameters of UHMWPE resin, including molecular weight, molecular weight distribution, particle size, and particle size distribution [4,5,6,7,8]. These parameters further alter the properties of UHMWPE fiber. In recent years, extensive research has been conducted on the relationship between UHMWPE resin parameters and fiber properties. Li et al. [9] investigated the impact of resin particle size and distribution on the swelling behavior of UHMWPE resin, further exploring the correlation between swelling characteristics and fiber quality. Huang et al. [5] studied the relationship between the properties of various UHMWPE resins and their spinning performance, discovering that a narrower range of particle size distribution was more conducive to dissolution. Zhang et al. [6] found that UHMWPE resins characterized by compactness, regularity, and high sphericity exhibited more stable viscosity during dissolution. Wang et al. [7] demonstrated that an increase in the molecular weight of UHMWPE resin enhanced its solubility. Additionally, Zhang et al. [8] reported that the impact strengths of UHMWPE fibers produced from UHMWPE resin with smaller particle sizes were significantly higher. However, the existing literature primarily emphasizes modifying resin parameters to enhance mechanical properties and spinning performance of fibers. There is a notable lack of systematic investigation into the relationship between UHMWPE resin parameters and the consistency in fiber linear density.

In order to address this research gap, a series of UHMWPE fibers were produced through wet spinning using UHMWPE resins with varying parameters. The effects of molecular weight, molecular weight distribution, particle size, and particle size distribution of UHMWPE resins on the consistency of linear density and the mechanical properties of UHMWPE fibers were systematically investigated. Additionally, potential mechanisms underlying these effects are discussed. This study is significant for the production of high-consistency UHMWPE fibers.

## 2. Materials and Methods

### 2.1. Materials

The characteristics of UHMWPE resin are presented in Table 1; the catalyst system employed during resin production remained consistent, with the residual catalyst content was required to be ≤250 ppm to meet industrial specifications. Industrial white oil was purchased from Zhejiang Zhengxin Petroleum Technology Co., Ltd., China, ZheJiang Province, and light white oil were purchased from Jiangsu Wuyang Hydrocarbon Technology Co., Ltd., China, JiangSu Province. All the chemicals and reagents were of analytical grade and used without further purification. Sample e was established as the precursor material consistently employed in our current production phase.

### 2.2. Methods

#### 2.2.1. Preparation of UHMWPE Fiber

**Figure 1:**
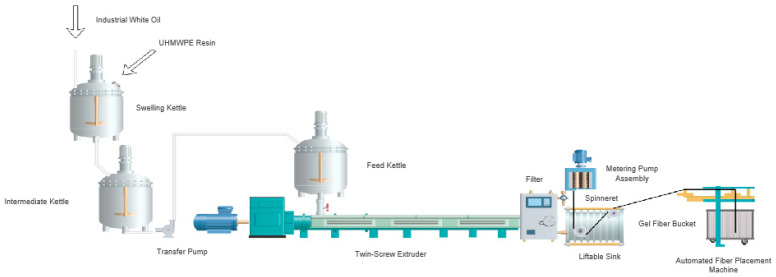
Schematic diagram of the pre-spinning process of UHMWPE fibers.

Figure 1 is the schematic diagram of the pre-spinning process of UHMWPE fibers, at the specified temperature, industrial white oil was blended with UHMWPE resin of varying parameters in a constant ratio between 7.0 and 10.0%, using a swelling kettle (Yancheng Daming Chemical Machinery Co., Ltd., China, JiangSu Province, 3.5 m^3^); the temperature range was maintained at 110–118 °C during processing. The resulting mixture was then directed to an intermediate kettle (Yancheng Daming Chemical Machinery Co., Ltd., China, JiangSu Province, 4 m^3^), followed by a feed kettle (Yancheng Daming Chemical Machinery Co., Ltd., China, JiangSu Province, 1.5 m^3^) and a twin-screw extruder (Jiangsu Chengmeng Equipment Co., Ltd., China, JiangSu Province 125X160603A), and the shear temperature in twin-screw processing was controlled within the range of 180–250 °C to optimize polymer chain alignment and material homogeneity. Subsequently, it passed through a metering pump and spinneret before being extruded into a 5–15 °C cooling water bath to produce cooling fibers. Finally, nascent fibers were obtained by drawing the cooled fibers at a constant speed of 6–15 m/min.

**Figure 2:**
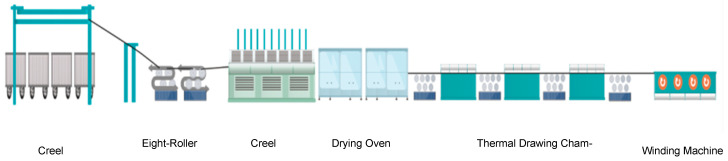
Schematic diagram of post-spinning process of UHMWPE fibers.

Figure 2 is the schematic diagram of the post-spinning process of UHMWPE fibers, after nascent fibers have attained phase equilibrium, the initial drawing process was conducted using an eight-roller drawing machine (Jiangsu Shenhe Technology Development Co., Ltd., China, JiangSu Province, SGJ-PE2), and the draw ratio was maintained within 3–5 times during the fiber processing stage, followed by an extractor (Jiangsu Shenhe Technology Development Co., Ltd., China, JiangSu Province, SCQ-PE2) filled with different concentration of light white oil and a drying oven (Jiangsu Shenhe Technology Development Co., Ltd., China, JiangSu Province, RFGZ-PE2). This was succeeded by ultra-multiple hot drawing utilizing a seven-roller drawing machine (Jiangsu Shenhe Technology Development Co., Ltd., China, JiangSu Province, QSG-PE2) in conjunction with a thermal drawing chamber (Jiangsu Shenhe Technology Development Co., China, JiangSu Province, Ltd., RFQS-PE2), the drawing temperature was maintained at 145–150 °C with a drawing ratio controlled within 20–30 times. Finally, the resulting UHMWPE fibers were collected using a winding machine (Jiangsu Rifa Machinery Technology Co., Ltd., China, JiangSu Province, SJ-FJ) with speed of 30–40 m/min.

#### 2.2.2. Characterization 

The weight average molecular weights (M_w_) and Z-average molecular weights (M_z_) of UHMWPE resins were performed at 150 °C on a Malvern PL-GPC-220 gel permeation chromatography (GPC). 1,2,4-trichlorobenzene was used as the eluent at a flow rate of 1.0 mL·min^−1^, narrowly dispersed polystyrene as the standard [8], and styrene-divinyl benzene (T-6000, 8.0 × 300 mm, 20 μm) as the chromatographic column. The molecular weight distribution was calculated as the ratio of M_z_ to M_w_, denoted as M_z_/M_w_. The intrinsic viscosities ([η]) of UHMWPE resins were obtained using a kinematic viscosity tester (Shanghai Changji Geological Instrument Co., Ltd., Shanghai, China, SYD-265E). The viscosity average molecular weights were determined with reference to the empirical formula (M = 55.64 × [η]^1.49^) [10].

The surface and cross-section of the UHMWPE fibers were coated with a 5 nm-thick layer of gold utilizing a Leica ACE600 sputtering machine. The particle morphology of UHMWPE resins was characterized using an EVO 10 scanning electron microscopy (SEM) [11], operated at an accelerated voltage of 10 kV. Finally, statistical analysis of the particle size distribution was conducted employing Nano Measurer and Origin software.

Fibers manufactured under identical process parameters and within the same production timeframe were classified into the same batch. The linear density of the same batch of UHMWPE fiber was measured [12] with a Uster yarn evenness tester (UT6-S800/SA), and the coefficient of variation (CV) of linear density between bobbins was calculated.

A fiber roll with a total length of approximately 37,500 m underwent quality inspection through sampling 1000 m segments for fiber linear density analysis; the consistency of the linear density within the bobbin was calculated using the coefficient of variation (CV) as the statistical metric. The linear density of fibers produced from UHMWPE resin with varying parameters was also measured [13] by a Uster yarn evenness tester (UT6-S800/SA).

A fabric strength tester (Wenzhou Darong Textile Instrument Co., Ltd., Wenzhou, China, YG(B)026H) with a stretching speed of 250 mm·min^−1^ was used to study the mechanical properties of UHMWPE fibers (length: 500 mm) at 25 °C [14], and at least three parallel tests were performed on each UHMWPE fiber.

Ballistic inserts fabricated from ultra-high-molecular-weight polyethylene (UHMWPE) fibers derived from different precursor resins under identical processing conditions were subjected to ballistic resistance evaluation [15]. This methodology systematically assesses both the structural uniformity of protective composites and their anti-penetration capabilities through controlled projectile impact tests.

## 3. Results and Discussion

### 3.1. Effect of Molecular Weight and Distribution on the Consistency of Linear Density in UHMWPE Fiber

#### 3.1.1. Effect of Viscosity Average Molecular Weight (M_η_) on the Consistency of Linear Density in UHMWPE Fiber

**Figure 3:**
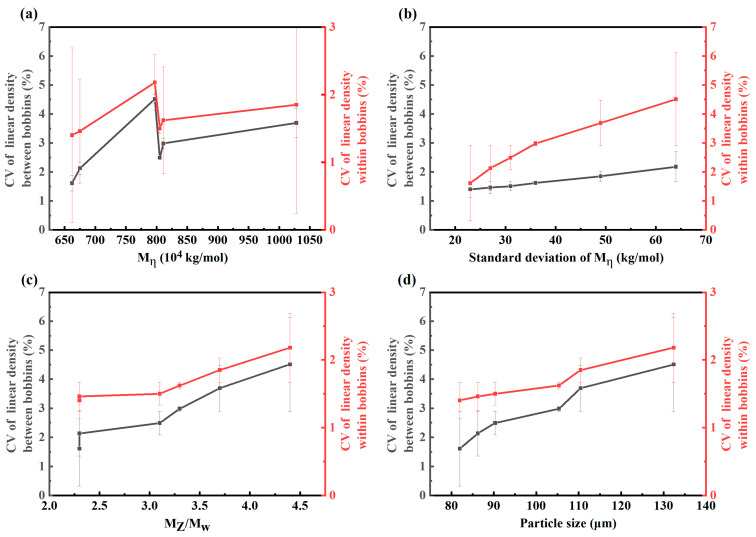
Effect of (**a**) viscosity average molecular weight (M_η_), (**b**) standard deviation of M_η_, (**c**) molecular weight distribution (the ratio of Z-average molecular weight to weight average molecular weight (M_Z_/M_w_)), and (**d**) particle size on the consistency of linear density in UHMWPE fiber.

As illustrated in Figure 3a, there was no significant correlation observed between the M_η_ of the resin and the coefficient of variation (CV) of linear density among bobbins. When the molecular weight was below eight million, the molecular chain length was relatively short, and the entanglement point density was low. During the gel spinning process, the melt exhibited shear-thinning behavior. At this time, the relaxation time of the molecular chains was short, and elastic turbulence was prone to occur when extruded through the spinneret orifices, resulting in non-uniform as-spun fibers. When the molecular weight reached eight million, the chain entanglement density reached an optimal value, forming a solid-like viscoelastic response. High-molecular-weight chains suppressed local flow instability through topological interlocking. Meanwhile, the solvation effect kept the melt viscosity within the spinning process window, enabling stable laminar flow extrusion. Excessively high entanglement density caused the melt to show a significant normal stress difference. An extrusion swelling effect occurred at the spinneret orifice exit, inducing a difference in the cooling rate between the fiber surface layer and the core layer. This led to the formation of a skin-core structure with delamination, resulting in a decrease in the uniformity of the as-spun fibers again.

#### 3.1.2. Effect of Molecular Weight Deviation and Molecular Weight Distribution on the Consistency of Linear Density in UHMWPE Fiber

As depicted in Figure 3b,c, a lower standard deviation of M_η_ and narrower molecular weight distribution (M_Z_/M_w_) of the resin were associated with a reduced CV of the linear density between and within UHMWPE fiber bobbin. This was due to the fact that during the spinning process, low-molecular-weight components dissolve faster, preferentially occupying the solvent and causing a sharp increase in solution viscosity. Meanwhile, high-molecular-weight components experience hindered dissolution due to solvent preemption by low-molecular-weight chains, leading to localized undissolved particles or concentration gradients. Using UHMWPE resin materials with narrow molecular weight distribution significantly reduced these dissolution disparities, ensuring superior solution homogeneity.

Conversely, employing UHMWPE resins with consistent molecular weights promoted homogeneity in the nascent fibers following twin-screw shearing. This uniformity facilitated ultra-multiple hot drawing [16], ultimately yielding high-consistency UHMWPE fibers.

### 3.2. Effect of Particle Size and Distribution on the Consistency of Linear Density in UHMWPE Fiber

#### 3.2.1. Effect of Particle Size on the Consistency of Linear Density in UHMWPE Fiber

As presented in Figure 3d, the smaller particle size of the resin corresponded to lower CV of linear density and higher consistency of the fiber. This phenomenon could be attributed to the fact that smaller particle size resins exhibited larger specific surface area and shorter solvent penetration paths, which accelerated the disentanglement process of molecular chains. The more consistent swelling rate reduced undissolved particles or concentration gradients caused by localized swelling discrepancies when processed through gel-spinning and super-drawing, and small particle size resins demonstrated a higher proportion of extended-chain crystals and narrower crystalline size distribution, directly contributing to the improvement in fiber uniformity.

In contrast, large-particle resin demonstrated an increase in dimensions and formed a high-viscosity swelling layer on their surfaces during the dissolution process. This characteristic impeded solvent penetration into the particles, leading to variations in solution viscosity that ultimately affected the consistency of fibers.

#### 3.2.2. Effect of Particle Size Distribution on the Consistency of Linear Density in UHMWPE Fiber

**Figure 4:**
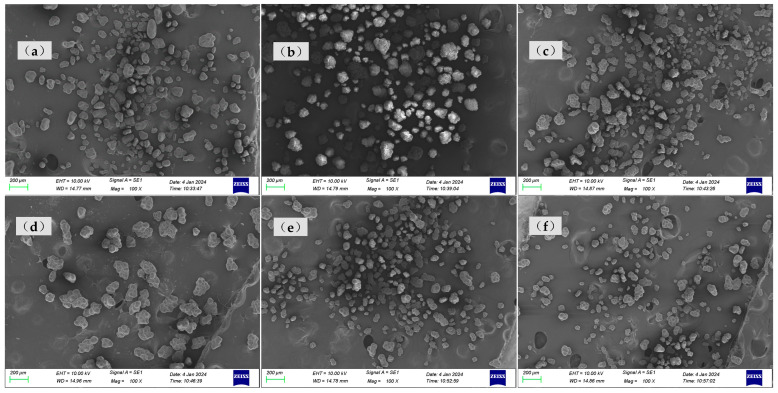
The SEM images of (**a**) sample a, (**b**) sample b, (**c**) sample c, (**d**) sample d, (**e**) sample e, and (**f**) sample f.

The SEM images of samples a–f are presented in Figure 4, and the corresponding particle size distributions are summarized in Table 2. As shown in Figure 4, sample A particles exhibited irregular shapes with rough surfaces, uneven edges, and significant size variations, showing notable differences in particle dimensions, while sample B particles displayed more rounded forms with relatively smooth surfaces, uniform size distribution, and concentrated particle dimensions. Sample c particles exhibited diverse morphologies with a wide size distribution, showing the presence of notably large particles. Sample d particles were predominantly irregular in shape with rough surfaces, characterized by relatively large particle sizes and a concentrated size distribution. Sample e particles displayed rounded morphologies with smooth surfaces and uniform particle size distribution. Sample f particles primarily demonstrated irregular shapes with rough surfaces, featuring a broad size distribution that included significantly large particles.

As illustrated in Table 2, narrower particle size corresponded to a lower CV of linear density and higher consistency of the fiber. This phenomenon could be attributed to the fact that a wide particle size of resins distribution increased the likelihood of larger particles settling during the swelling process [7,17]. Resins with a narrow particle size distribution exhibited more consistent swelling rates in solvents, thereby forming a spinning dope with reduced viscosity fluctuations. This minimized undissolved particles or concentration gradients in gel fibers caused by localized swelling differences. Simultaneously, during twin-screw shear processing, narrow-distribution resins demonstrated uniform responses to shear forces among particles, significantly reducing disparities in molecular chain disentanglement rates. This prevented the coexistence phenomenon observed in broad-distribution resins where fine particles underwent premature disentanglement while larger particles experienced delayed disentanglement. The resultant homogeneous spinning solution stabilized shear rates during extrusion processes and reduced diameter fluctuations in gel fibers.

### 3.3. Effect of UHMWPE Resin Properties on Mechanical Properties of UHMWPE Fiber

**Figure 5:**
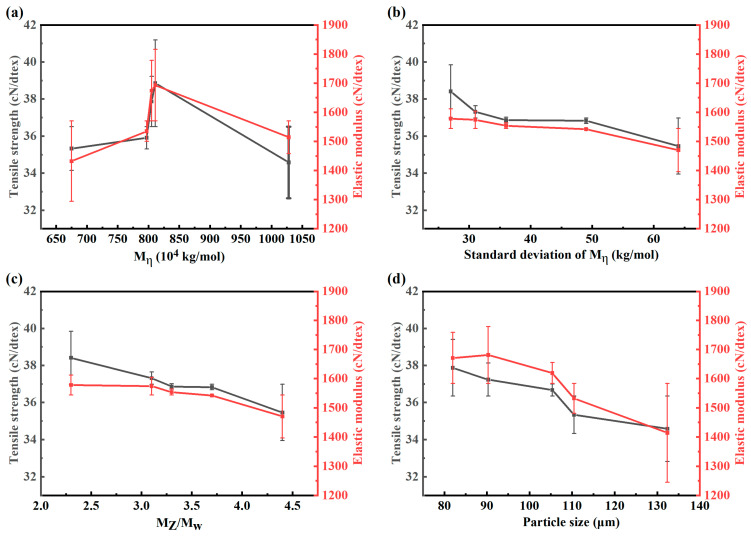
Effect of (**a**) M_η_, (**b**) standard deviation of M_η_, (**c**) M_Z_/M_w_, and (**d**) particle size on mechanical properties of UHMWPE fiber.

From Figure 5, it can be observed that molecular weight and its distribution, as well as particle size and its distribution, all exerted influences on the strength and modulus of UHMWPE fibers, with molecular weight and particle size demonstrating more pronounced effects.

#### 3.3.1. Effect of M_η_ on Mechanical Properties of UHMWPE Fiber

As illustrated in Figure 5a, tensile strengths and elastic modulus of the fiber increased and then decreased with increasing molecular weight of the resin. The increase in molecular weight of UHMWPE led to significant elongation of molecular chains and an exponential rise in entanglement point density. When the molecular weight was <8 × 10^6^, during gel spinning, the twin-screw shearing effect could effectively disentangle molecular chains, forming a sol system with moderate entanglement. At this stage, the molecular chains possessed sufficient length to ensure stretch-induced orientation potential while avoiding excessive entanglement that would hinder disentanglement [18,19]. However, when the molecular weight exceeded 8 × 10^6^, the chain entanglement density surpassed the critical threshold. Even under high shear stress, complete disentanglement became unachievable, leaving residual entanglement points that acted as stress concentration sites during stretching. These residual defects ultimately compromised the mechanical performance of UHMWPE fibers.

#### 3.3.2. Effect of Molecular Weight Deviation and Molecular Weight Distribution on Mechanical Properties of UHMWPE Fiber

As depicted in Figure 5b,c, tensile strengths and elastic modulus of the fiber increased with decreasing standard deviation of M_η_ or M_Z_/M_w_ of the resin. This might be because when the molecular weight distribution is relatively narrow, the lengths of the molecular chains are relatively uniform. During the gel spinning process, it is easier to achieve synchronous stretching-induced orientation, resulting in a high degree of crystallinity and a uniform lamellar crystal structure. As the standard deviation increases, due to the fast segment relaxation rate of the low-molecular-weight components, it is difficult for them to be oriented synchronously with the high-molecular-weight components. This causes the low-molecular-weight chains to break preferentially during stretching, forming microcracks. The entanglement point density of the high-molecular-weight chains decreases, and the stress transfer efficiency declines. When the standard deviation reaches 40–50 kg/mol, a dynamically balanced entanglement–slip system may form. That is, the low-molecular-weight chains absorb energy through chain-end slippage, while the high-molecular-weight chains maintain the skeletal load, slowing down the deterioration trend of the mechanical properties.

#### 3.3.3. Effect of Particle Size on Mechanical Properties of UHMWPE Fiber

As shown in Figure 5d, tensile strengths and elastic modulus of the fiber increased with decreasing particle size of the resin. This phenomenon could be attributed to the fact the reduction in particle size of UHMWPE resin decreases the density of physical entanglement points between molecular chains. During sol-gel spinning, smaller resin particles more readily form a uniform entanglement network in the solvent. The shear forces generated by twin-screw mixing promote molecular chain disentanglement. Higher degrees of disentanglement facilitate the achievement of highly oriented molecular chains along the fiber axis during subsequent multi-stage hot stretching processes, thereby enhancing the tensile strength and modulus of the fiber.

#### 3.3.4. Impact of UHMWPE Fiber Consistency on Ballistic Product Performance

The ballistic testing was conducted at room temperature with the following parameters: firing distance of 5 m, velocity measurement point located 3 m from the muzzle, and target placed 2 m beyond the velocity measurement point. The bulletproof plate (400 mm × 400 mm) test results documented three rounds successfully penetrated the armor, while three rounds were effectively stopped by the protective material. This dual-outcome reflects the plate’s variable performance under standardized penetration testing conditions.

As shown in Table 3, the higher the uniformity of UHMWPE fibers was, the better and more consistent the ballistic performance of armor plates was. This phenomenon can be attributed to the following mechanisms: (1) Highly uniform UHMWPE fibers exhibit a well-organized extended-chain crystal structure with axially aligned molecular chains. This structural characteristic endows the fibers with superior tensile strength and modulus, significantly enhancing energy absorption efficiency during projectile penetration. (2) In armor plates made of uniform fibers, the homogeneous distribution system allows stress waves to propagate through the isotropic medium with minimal energy dissipation. This efficient energy transfer accelerates the conversion of projectile kinetic energy into elastic strain energy within the fiber matrix. (3) Improved fiber uniformity reduces internal defects in ballistic products. Fewer defects prevent localized stress concentrations and premature structural failure, thereby maximizing the material’s inherent energy dissipation capabilities.

## 4. Conclusions

This paper systematically explored the impact of M_η_ and its deviation, molecular weight distribution, and particle size (and its distribution) of the UHMWPE resin on the consistency of linear density and mechanical properties of UHMWPE fibers. Compared with previous studies, this work systematically investigated the impact of UHMWPE raw resin properties on fiber uniformity, thereby addressing a critical research gap in this field. Key conclusions are summarized as follows:

i. Lower standard deviation of M_η_ or narrower M_Z_/M_w_ of the resin were associated with a higher consistency of fiber linear density. Smaller particle size or narrower particle size of the resin corresponded to a higher consistency of fiber linear density. There was no significant correlation between M_η_ of the resin and the consistency of fiber linear density.

ii. With the increase in the molecular weight of the resin raw material, the strength and modulus of UHMWPE fibers first increased and then decreased. A narrower molecular weight distribution range of the resin led to a higher strength and modulus in UHMWPE fibers. As the resin particle size increased, the strength and modulus of UHMWPE fibers decreased. Additionally, molecular weight and particle size exhibited a more significant impact on strength and modulus compared to other factors.

iii. The better the fiber consistency was, the better the protective performance of ballistic products was.

## Figures and Tables

**Table 1:** The manufacturers and characteristics of UHMWPE resin.

UHMWPE Resin NO.	M_η_ * (10^4^ kg/mol)	M_z_ * (10^4^ kg/mol)	M_w_ * (10^4^ kg/mol)	Average Particle Size (μm)
Sample a	662	315	137	82.0
Sample b	675	767	332	86.2
Sample c	797	465	107	90.3
Sample d	805	507	165	110.4
Sample e	811	611	183	105.4
Sample f	1028	727	196	132.4

* M_η_: viscosity average molecular weight, M_z_: Z-average molecular weight, M_w_: weight average molecular weight.

**Table 2:** Effect of particle size distribution on the CV of linear density.

UHMWPE Resin NO.	Particle Size Distribution	CV of Linear Density (%)	
		Between Bobbins	Within Bobbins
Sample a	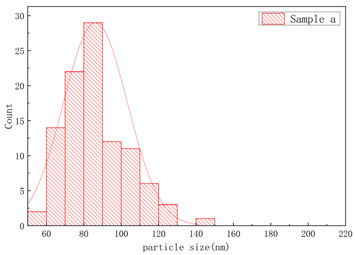	1.61	1.4
Sample b	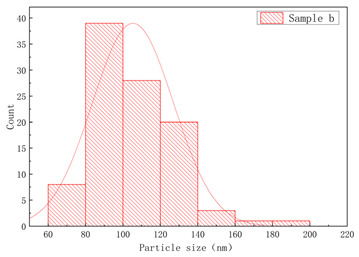	2.13	1.46
Sample c	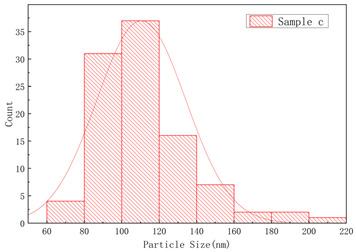	4.51	2.18
Sample d	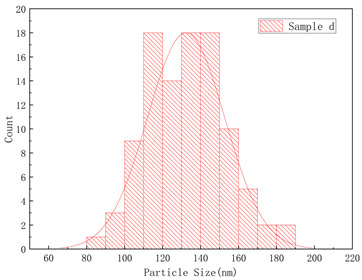	2.49	1.50
Sample e	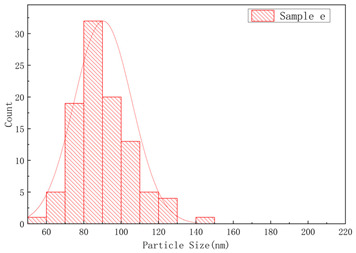	2.98	1.62
Sample f	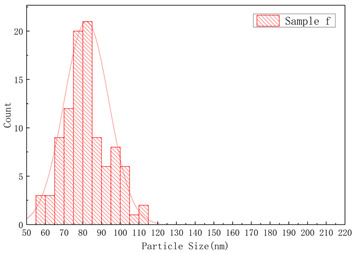	3.69	1.85

**Table 3:** Impact of UHMWPE fiber consistency on ballistic product performance.

Test Number	Bullet Speed (m/s)	
	Sample b	Sample c
1	642.00	599.00
2	648.00	611.00
3	638.00	616.00
4	665.00	625.00
5	653.00	588.00
6	666.00	594.00
Variance (m^2^/s^2^)	135.49	200.22
Value of V50 (m/s)	627.72	582.95

## Data Availability

The original contributions presented in this study are included in the article. Further inquiries can be directed to the corresponding author.

## References

[1] Gibson A.G., Ward I.M. (1980). The manufacture of ultra-high modulus polyethylenes by drawing through a conical die. J. Mater. Sci..

[2] Hoogsteen W., Brinke G.T., Pennings A.J. (1988). DSC experiments on gel-spun polyethylene fibers. Colloid Polym. Sci..

[3] Smith P., Lemstra P.J., Pijpers J.P.L., Kiel A.M. (1981). Ultra-drawing of high molecular weight polyethylene cast from solution: III. Morphology and structure. Colloid Polym. Sci..

[4] Xia Y., Ni J., Wang Y., Guo Z., Wang X. (2018). The cutting-edge technology of UHMWPE fiber from DSM. Text. Sci. Res..

[5] Huang X. (2019). Research on the Properties and Spinning Performance of Different UHMWPE Raw Materials. Synth. Fiber China.

[6] Zhang W., Wu Z., Wang X. (2020). The relationship between particle morphology and structural properties of ultra-high molecular weight polyethylene resin. Polym. Mater. Sci. Eng..

[7] Wang X., Zhang Y., Wu X., Xu J. (2011). Study on the swelling properties of ultra-high molecular weight polyethylene resin. Synth. Fiber China.

[8] (2018). Standard Practice for Reporting Results of Examination and Analysis of Deposits Formed from Water for Subsurface Injection.

[9] Li B. (2013). Review of the award-winning achievements of the first prize of science and technology of the China National Textile and Apparel Council in 2013 (“Textile Light”) (II). China Text Lead..

[10] (2010). Plastics—Determination of the Viscosity of Polymers in Dilute Solution Using Capillary Viscometers—Part 3: Polyethylenes and Polypropylenes.

[11] (2021). Nanotechnologies—Measurements of Particle Size and Shape Distributions by Scanning Electron Microscopy.

[12] (2008). Chemical Fibers—Test Method for Linear Density of Filament and Textured Yarns.

[13] (2021). Textiles—Determination of Yarn Linear Density (Mass per Unit Length) Variability by Capacitance Testing.

[14] (2005). Test Method for Tensile Properties of High-Tenacity Chemical Filament Yarns.

[15] (2011). Ballistic Materials and Products—V50 Test Method.

[16] Zhang H., Zhao S., Yu X., Xin Z., Ye C., Li Z., Xia J. (2019). Nascent Particle Sizes and Degrees of Entanglement Are Responsible for the Significant Differences in Impact Strength of Ultrahigh Molecular Weight Polyethylene. J. Polym. Sci. Part B-Polym. Phys..

[17] Yang B., Jiang W., Wang J., Yang Y. (2005). Study on particle size distribution model of polyethylene particles in fluidized bed with gas phase. J. Chem. Eng. Chin. Univ..

[18] Romano D., Tops N., Andablo-Reyes E., Ronca S., Rastogi S. (2014). Influence of polymerization conditions on melting kinetics of low entangled UHMWPE and its implications on mechanical properties. Macromolecules.

[19] Ma L., Wang X., Yu J., Wang Y., Hu Z., Zhu J. (2017). Degradation behavior of UHMWPE gel in spinning and its Effect on fiber properties. Synth. Fiber Ind..

